# Physicochemical, Sensory and Nutritional Properties of Foods Affected by Processing and Storage

**DOI:** 10.3390/foods10122970

**Published:** 2021-12-02

**Authors:** Sidonia Martínez, Javier Carballo

**Affiliations:** Food Technology Area, Faculty of Sciences, University of Vigo, 32004 Ourense, Spain; carbatec@uvigo.es

Due to their chemical composition and physicochemical characteristics, most foods are very perishable and are easily altered by physical, chemical and biological agents. To increase their shelf-life and thus enable their transport and storage, it is necessary to carry out specific technological treatments that protect them from deterioration agents. In other cases, technological treatments are applied with a sanitizing purpose, destroying pathogens, or with the aim of diversification, seeking to obtain from a concrete raw material a wide range of different products in order to satisfy the diverse needs and demands of consumers. In any case, sanitizing or diversification treatments are almost always accompanied by an increase in shelf life. Treatments can also be intended to condition food, making it more suitable for consumption. Food processing is therefore a common and necessary activity in order to obtain the wide range of foods that we know, safe and with their diverse sensory and nutritional profiles. However, sometimes these processes have negative effects that result in a decrease in nutritional value or in the alteration of sensory properties. Even sometimes compounds negative for the health of the consumer are generated. It is therefore necessary to gather as much knowledge as possible about the effects of technological treatments on food components in order to optimize these treatments and to minimize their negative effects and achieve food of the highest quality and wholesomeness.

The articles included in the present special issue show important and interesting advances and new approaches in this field.

Canning is one of the most important procedures of food preservation. From the initial works of Nicolas Appert [1], extensive and ongoing research has been carried out aimed at improving the process and focused on the development of new materials for packaging, on optimizing heat treatments and their adaptation to the characteristics of the food to be preserved and on the study of the effect of the filling medium to select the most appropriate in each case.

Marine foods are traditionally preserved by canning, with canned fish and seafood being products of proven quality that are very popular and well-established among consumers. Constituents and organoleptic properties of marine foods have different thermal sensitivity and according to this, different detrimental effects were reported in the literature. In this special issue, three articles make interesting contributions within this research topic. Aubourg et al. [2] studied the effect of the duration of the previous chilling period (0, 4 and 9 days) and of the use of an aqueous extract of the macroalga *Fucus spiralis* in the brine-packaging medium (final contents of 0.00, 3.50, 10.50 and 21.00 mg extracted alga/mL packaging medium) on the chemical changes related to quality registered after 3 months of storage in a canned underutilized mackerel species (*Scomber colias*). Increased chilling times increase the free fatty acid content, but the use of the alga extract reduced this content. An increased chilling time led to the increase of the values of the lipid oxidation indexes and the presence of the alga extract had an uneven effect on these parameters. Trimethylamine content markedly increased after the sterilization process and no effects of the chilling time or of the alga extract addition were observed.

Gómez-Limia et al. [3] evaluated the effects of the steps of the canning process (raw, after frying, after sterilization and after 2 and 12 months of storage) and of different filling media (sunflower oil, olive oil, or spiced olive oil) on the free amino acid and biogenic amine content of the European eel (*Anguilla anguilla*). Relative to raw samples, all steps in the manufacture and storage significantly affected the free amino acid content of eels, this content being also affected by the type of oil used in the frying process and as the filling medium. The step of the canning process, the type of oil used in the frying process or as filling medium and the storage time also affected the contents of the different biogenic amines. The authors reported very low biogenic amine contents and an absence of histamine in all samples. The low biogenic amine index observed indicate the good quality of canned eels.

Gómez-Limia et al. [4] also studied the effects of the steps of the canning process and of different filling media on the oxidative stability and antioxidant activity in canned eels. After the same steps as in the previous study and testing the same oils as filling medium, the authors assessed the oxidation parameters (acidity, peroxide value and thiobarbituric acid reactive substances), antioxidant capacity and total phenolic and vitamin E contents. Despite the losses, the canning process and subsequent storage preserved a great part of the antioxidant capacity and vitamin E content of the filling medium, which is of great interest to the consumer.

Pet food can also appear in the markets canned. Most of these goods are products consisting of meat and water containing binding/structural ingredients, commercially sterilized and similar to stews and pâtés in appearance. Hydrocolloids are commonly used in canned pet food for firmness control, but their functional effects have not been quantified in this food format. Dainton et al. [5] determined the effects of select hydrocolloids (1% dextrose and 0.5% guar gum with 0.5% of either dextrose, kappa carrageenan, locust bean gum, or xanthan gum) on batter consistency, heat penetration and the texture of canned pet food. The authors showed that hydrocolloids influenced heat penetration, probably due to differences in batter consistency and affected finished product texture.

Dehydration is one of the most ancient and effective method for food preservation. The reduction of the water content hinders the activity of the agents of deterioration, both of the microorganisms and enzymes. At the same time, the elimination of water reduces the weight and volume of the food and facilitates its package, transport and storage. Additionally, dehydration provides particular organoleptic characteristics, creating new foods in some cases [6]. In this special issue, three different articles reported the effects of dehydration achieved by different procedures on several food constituents and characteristics.

Saffron is the dried stigmas of *Crocus sativus*, its high value being due to the color, flavor and aroma. The dehydration procedure used affects the main metabolites of saffron that are responsible for these organoleptic characteristics and that define the quality of the product. García-Blázquez et al. [7] studied the effect of microwave dehydration on saffron main metabolites (picrocrocin, safranal and crocetin esters) from *Crocus sativus* L. stigmas at three determinate powers and different time lapses. A control of saffron dehydrated using the traditional dehydration called “toasting” in which stigmas are put on a sieve with a silk bottom placed over a heating source was obtained and analyzed. The results reported suggest that microwave dehydration is a suitable process for obtaining high quality saffron, 800 W with 6 lapses of 20 s being the best conditions among those studied.

Fresh wet noodles (FWN) have high water and nutrient contents that favor microbial growth. Therefore, a preservation treatment is necessary in order to extend the shelf-life without decreasing the organoleptic and nutritional quality. Several treatments such as pasteurization, pH decrease, natural or chemical preservatives, heat, irradiation, non-thermal processing technologies, ozone water treatment, modified atmosphere packaging, hurdle technology, etc., have been tried for fresh wet noodle preservation with unequal results. Drying is of course another valid alternative. The industrial drying process of noodle products was often at low temperature with divided steps, and the humidity of the hot air was usually taken into consideration for fine dried noodles. The relative humidity, and the temperature of the hot air, should be controlled during the drying process for both semi-dried noodles and fresh wet noodles because heat treatment with excessive temperature and humidity may result in the over gelatinization or melting of starch and the generation of cracks or chaps in fresh noodles, thus deteriorating the noodle quality. The humidity-controlled dehydration (HCD) at moderate temperatures could be a successful technique to control the microbial growth in fresh wet noodles. Xing et al. [8] investigated the effects of HCD treatment using different temperatures, relative humidity and treatment times on the total plate count, the shelf-life and qualities (color, degree of gelatinization, cooking and textural properties and sensory attributes) of FWN. Data reported by these authors showed that HCD reduced the initial microbial load on the fresh noodles and extended their shelf-life by up to 14–15 days under refrigerated (10 °C) storage. The lightness (L*) values, the apparent stickiness and the cooking properties of the noodle body were improved by HCD while good sensory and texture quality of noodles were still maintained after the dehydration process.

Pear (*Pyrus communis* L.) can be maintained for long periods of time (close to a year) under controlled atmosphere. However, long-term storage in these conditions can promote deterioration processes such as internal browning and superficial scald, negatively influencing the consumer acceptability. Drying can be an alternative to increase the shelf-life of pears with the added benefit of reducing transportation and storage costs, also offering new ways of presentation for consumers. Conventional hot air drying is widely used in the food industry, but it can promote the partial degradation of nutrients and undesirable changes in color, appearance and structural properties. Pre-treatments with modern technologies such as microwaves or ultrasound can accelerate the drying process, reducing drying time, enhancing mass transfer phenomena, preserving functional components, inactivating enzymes and improving rehydration characteristics. However, these pre-treatments can also have negative effects on the quality of the final product if the application conditions are not suitable. Therefore, more research is needed to stablish the optimal conditions for the application of the pre-treatments. In this context, Önal et al. [9] evaluated the effect of drying temperature and innovative pre-treatments (microwave and ultrasound) on drying behavior and quality characteristics, such as color, total phenolic content and antioxidant activity of “Rocha” pear, a traditional Portuguese cultivar. The authors concluded that ultrasound application is a promising technology to obtain healthy/nutritious dried “Rocha” pear snacks.

Fermentation followed by drying is a common combined procedure for obtaining the most reputed meat and dairy products. Dry-fermented sausages are widely manufactured and consumed meat products, deeply rooted in the culture and tradition of several regions and countries and with outstanding social and economic relevance. Most of the dry-fermented sausage varieties are manufactured in small-scale production plants following traditional procedures with scarce degree of mechanization and with limited control of the quality and safety of the final product. As a result, products are very heterogeneous in their organoleptic characteristics which hinders in many cases the loyalty of consumers and expansion to markets far from their areas of origin. The implementation of systems of control for the whole dry-fermented sausage process, ensuring the safety and quality of the product, would be of use to overcome these issues. However, due to the particularities of the producers, such a control system should be simple, cheap and easily implemented. Exploring the possibilities of one of these control systems, González-Mohino et al. [10] studied the ability of a MicroNIR device to monitor the dry fermented sausage process with the use of multivariate data analysis. Thirty sausages were manufactured, subjected to dry fermentation and studied into four main stages of the production process (raw material, end of fermentation, end of intense drying and final product). The authors performed physicochemical (weight lost, pH, moisture content, water activity, color, hardness and thiobarbiruric reactive substances analysis) and sensory (quantitative descriptive analysis) analysis of samples on different steps of the ripening process. Near-infrared (NIR) spectra were taken throughout the process at three points of the samples. The results of multivariate data analysis showed the ability to monitor and classify the different stages of ripening process (mainly the fermentation and drying steps). Based on their results, the authors concluded that a portable NIR device is a nondestructive, simple, noninvasive, fast and cost-effective tool with the ability to monitor the dry fermented sausage processing and to classify samples as a function of the stage. This device constitutes a feasible decision method for sausages to progress to the following processing stage.

Irradiation is one of the classical procedures for food preservation [11] based on the ability of ionizing radiation to destroy the biological agents of food spoilage, microorganisms and enzymes. Despite their limited penetration, electron beam irradiation (E-beam) has a widely demonstrated effectiveness for food preservation or sanitation. Rice (*Oryza sativa* L.) storage is largely affected by the moisture content. Rice with high moisture (HM) content has better taste but is difficult to store. Farmers generally use sunlight drying to reduce the moisture content of rice and to extend storage time. However, although moisture reduction extends rice shelf-life, it will also reduce the rice taste by increasing hardness. Therefore, another procedure for rice preservation will be of use. E-beam irradiation could be used for this purpose, but before exploiting this possibility, it would be interesting to see the effect of E-beam irradiation on the quality properties of rice. In a study included in this special issue, Pan et al. [12] applied low-dose electron beam irradiation (EBI) in newly harvested HM rice and in dried rice and studied the effect of the irradiation on the cooking quality and moisture migration of irradiated rice and on the thermodynamics and digestion properties of the isolated starches. Overall, low-dose EBI had little effect on the properties of rice. High moisture rice showed superior quality and taste, whereas low moisture rice exhibited superior nutritional quality. Therefore, low-dose EBI seems to be more effective than other techniques for rice preservation.

Food spoilage can also be prevented or slowed by using preservatives that delay microbial growth or slow spoilage reactions such as oxidation of food components. However, consumers are increasingly reluctant to chemical additives, which makes it necessary to replace traditional preservatives with natural substances that have similar functions. Natrella et al. [13] essayed the use of different preservatives to avoid the formation of undesirable volatile organic compounds (VOC) in stracciatella, a traditional Italian cream cheese. Samples of cheese were prepared by adding two different preservatives (sorbic acid and an olive leaf extract) alone or combined. Their effect on flavor preservation during the refrigerated storage of cheese was investigated by chemical, microbiological and sensory analyses. The best chemical and sensory results were obtained in the samples containing sorbic acid, alone or in combination with leaf extract, demonstrating a significant shelf-life extension. According to the results, the use of the olive leaf extract, at the concentration tested (400 mg/kg), seemed to be interesting only in the presence of sorbic acid.

Some compounds present in foods have particular (i.e., functional, bioactive, etc.) properties and it is desired to obtain them in purity for their inclusion in the formulation of other foods. In other cases, some compounds have negative effects and should be removed without further ado. Sometimes, therefore, food processing is aimed at the extraction of molecules present in its composition. Extraction processes have different degree of complexity and involve different techniques and are based on the chemical and physical characteristics of the components to be extracted. Aqueous extraction of carob kibbles is the most important step in the production of carob juice and carob molasses. Improving the theoretical yield in sugars during organic solvent-free aqueous extraction is of prime interest to the food industry. However, collateral extraction of phenolic compounds must be monitored as it influences the functional and sensory profile of carob juice. In a study present in this special issue, Antoniou et al. [14] examined the impact of source material, kibble size, temperature and duration on the efficiency of aqueous extraction of sugars and phenolics by conventional heat-assisted (HAE) and ultrasound-assisted (UAE) methods. Authors observed that source material was the most influential factor determining the concentration of phenolics extracted by either method. Disproportionate extraction of phenolics over sugars limits the use of heat-assisted extraction to improve sugar yield in carob juice production and may shift the sensory profile of the product toward astringency. However, prolonged extraction at near ambient temperature can improve sugar yield, keeping collateral extraction of phenolic compounds low. Authors concluded that ultrasound agitation constitutes an effective procedure of extracting sugars from powder-size kibbles and that the industrial application of both methodologies depends on the targeted functional and sensory properties of carob juice.

The term “marinade” is mainly used to refer a mixture of ingredients, in a liquid solution or powder form that is applied to raw foods to improve their organoleptic characteristics [15]. Marinating by food immersion or injection using solutions of different compounds (i.e., sauces, herbs, spices, organic acids, etc.) is a common practice for food conditioning before cooking or direct consumption.

Marinating can be used for meat tenderization by including a tenderizing agent in the liquid that is injected or in which the meat is submerged. Bromelain is a proteolytic enzyme widely essayed and used for meat tenderization. With the aim of meat tenderization by marinating, Santos et al. [16] added in marinades dehydrated pineapple by-products enriched in bromelain using a hydrostatic pressure treatment. Steaks from the silverside beef cut characterized as harder and cheaper were immersed in marinades containing dehydrated and pressurized pineapple (*Ananas comosus* L.) by-products that corresponded to a bromelain concentration of 0–20 mg tyrosine/100 g meat, 0–24 h time, according to the central composite factorial design matrix. After marinating, samples were characterized in terms of marination yield, pH, color and histology. Next, samples were cooked in a water-bath, stabilized and analyzed for cooking loss, pH, color, hardness and histology. Marinating times of 12–24 h and bromelain concentrations of 10–20 mg tyrosine/100 g meat reduced pH and hardness, increased marination yield and resulted in a lighter color. Meat hardness decreased by 41%, although refrigeration was not an optimal temperature for bromelain activity. Authors concluded that the use of pineapple by-products in brine allowed for the tenderization of lower commercial value steak cuts.

Marinating may be combined with other seasoning/conditioning methods for optimal results. In order to select the best processing conditions to obtain chicken meat with the highest sensory quality, Cho and Choi [17] after defrosting using different procedures (room temperature, running tap water, or high-frequency defroster) marinated chicken meat (leg and breast) with herbal extract solutions (bay leaves, coriander powder, fennel whole, thyme whole, cumin seeds, basil whole, basil powder, or star anise). Next, marinated meats were treated with superheated steam and then smoked with wood sawdust from different species (oak, apple, chestnut, walnut or cherry). Sensory evaluation was performed after each processing step. The products were also analyzed for fatty acids and nutrients (moisture, ash, salinity, calories, sodium, carbohydrates, sugars, dietary fiber, crude fat, trans fat, saturated fat, cholesterol, crude protein, calcium, iron, potassium and vitamin D), along with storage tests under different conditions monitored by microbiological, chemical and sensory analyses. High-frequency defrosting was the best method in terms of drip loss and thawing time. Bay leaves for marinating and oak wood for smoking were selected as the best materials for higher sensory scores. Optimal superheated steam conditions that showed the highest overall acceptance were 225 °C during 12 min and 20 s for leg meat and 223 °C during 8 min and 40 s for breast meat. The final meat products possessed good nutritional composition and no severe sensory spoilages were detected during storage.

Cooking is the most widely used procedure for food conditioning before consumption. Cooking can have positive or negative impact on the organoleptic characteristics and nutritional value of foods. Therefore, cooking method and parameters should be adapted to each raw material for optimal quality of cooked products. Nawaz et al. [18] evaluated the effect of various cooking methods (frying, baking and microwave cooking) on quality, structure, pasting, water distribution and protein oxidation of fish meat-based snacks. The findings suggest the endorsement of baking and microwave cooking for obtaining quality, safe and healthy snacks.

Some foods have a seasonal production but their demand and consumption is produced throughout the year. In other cases, it is very difficult to adapt the rhythm of production to that of consumption. Therefore, food storage is a necessary operation. Some methods of food preservation (canning, dehydration, irradiation) destroy or inhibit biological spoilage agents and allow long-term storage of food. However, such preservation procedures modify the organoleptic properties of foods, significantly distancing them from the sensory attributes that the same foods have in the fresh state. Some foods therefore should be stored in the fresh state and it is necessary to adopt other strategies to minimize their alteration during storage. In such cases, it is extremely important to know the changes that occur during storage to try to avoid or minimize them. Low temperatures retard microbial growth to a different degree depending on the nature of the microorganisms and slow down enzymatic reactions. That is why the storage of food at low temperatures is a useful and widely adopted procedure for prolonging the shelf-life of foods by minimally altering their organoleptic characteristics and nutritional value. In this special issue, several works studied the physical and chemical changes experienced by foods during storage.

The extra virgin olive oil (EVOO) is a typical food in which the extent and conditions of storage may affect its stability and quality. Mousavi et al. [19] evaluated the effects of different conditions of storage (ambient, 4 °C and −18 °C temperatures, and argon headspace) on three EVOOs (with low, medium and high phenol contents) over 18 and 36 months, through the analyses of the main metabolites at six time points. The organoleptic attributes of the oil samples were evaluated at all time points by a panel of tasters. The results showed that low temperatures are able to maintain all three EVOOs within the legal limits established by the current EU regulations for most compounds up to 36 months. The best temperature for conservation during 36 months was 4 °C, but −18 °C represented the optimum temperature to preserve the organoleptic properties. This study provided new insights that should guide EVOO manufacturers and traders to apply the most efficient storage methods to maintain the characteristics of the freshly extracted oils for a long conservation time.

The blueberry is widely cultivated throughout the world and its production has increased considerably in recent years. Yan et al. [20] studied the changes of volatile composition and other quality traits (weight loss, decay index, firmness and physicochemical parameters) of blueberry “garden blue” during postharvest storage. Odor was also evaluated by a sensory panel. Blueberries were packaged in vented clam-shell containers and stored at 0 °C for 0, 15 and 60 days, followed by storage at room temperature (25 °C) for up to 8 days for quality evaluation. The results of this work proved that cold storage was a dependable way to maintain the quality of blueberry. Nevertheless, a flavor deterioration was observed during subsequent shelf life.

Due to seasonality and short shelf life, approximately 50% of blueberries produced worldwide are processed, transforming them mainly into juice and dry fruits. Most of the blueberries used for juice production or drying are frozen fruits. During frozen storage and transportation, blueberries might be subjected to several freeze–thaw (FT) cycles. Understanding the changes in frozen blueberries during repeated FT cycles is essential for the processing of blueberry products because FT treatment will affect the qualities and flavors of the final food products. With this aim, Zhu et al. [21] studied the effects of FT cycles on the juice properties and aroma profiles and the hot-air drying kinetics of frozen blueberry. The authors reported that after FT treatment, the juice yield increased while pH and total soluble solids of the juice keep unchanged. The total anthocyanins contents and DPPH antioxidant activities of the juice decreased by FT treatments. The electronic nose showed that FT treatments significantly change the aroma profiles of the juice. The authors observed that one FT treatment can shorten the drying time by about 30% to achieve the same water content. Undoubtedly, the results of this work will be of use for the processing of frozen blueberry into juice or dried fruits.

In order to exploit the functional properties of fresh beetroot throughout the year, it is essential to maintain the health-benefiting compounds. With this purpose, Yasaminshirazi et al. [22] studied the impact of cold storage on bioactive compounds and their stability of beetroot. In their work, thirty-six beetroot genotypes collected from two field experiments, which were conducted under organic conditions, were evaluated regarding their content of total dry matter, total phenolic compounds, betalain, nitrate and total soluble sugars. Samples were analyzed directly after harvest and after cold storage periods of one and four months. The outcome of this study revealed a significant influence of genotype on all measured compounds. Furthermore, significant impacts were shown for storage period on total dry matter content, nitrate and total phenolic compounds. Therefore, the authors concluded that selection of the suitable genotype based on the intended final use is recommended to retain the quality of the beetroot for an extended time after harvest.

Finally, in this special issue, Lin et al. [23] studied the moisture migration, protein oxidation, microstructure and the physicochemical characteristics of Atlantic mackerel (*Scomber scombrus*) during storage at 4 °C and 0 °C. A slightly continuous decrease in the content of water and a certain degree of protein oxidation were observed over the course of storage. The storage process also caused changes in the secondary structure of proteins, the contraction and fracture of myofibrils and the granulation of endolysin protein. In addition, the drip loss, total volatile basic nitrogen content, thiobarbituric acid-reactive substances value and yellowness (b*) value significantly increased with the storage time.

It is evident that in recent years, much progress has been made in generating knowledge in this field. However, the chemical diversity of food components, the complexity of the interactions and effects that occur in such components as a consequence of the environmental conditions established during the different technological processes applied and during storage, and the requirements of consumers who increasingly demand safer food with higher organoleptic and nutritional quality make even more research necessary in order to achieve more efficient, economical and sustainable processes.
